# Supplementary material for: Bisphenols A and S Alter the Bioenergetics and Behaviours of Normal Urothelial and Bladder Cancer Cells
Source: Cancers (Basel). 2022 Aug 19;14(16):4011. doi: 10.3390/cancers14164011 (PMC9406715; doi:10.3390/cancers14164011)
Supplement: Supplementary file 1 [file cancers-14-04011-s001.zip › cancers-1847190-supplementary.pdf]

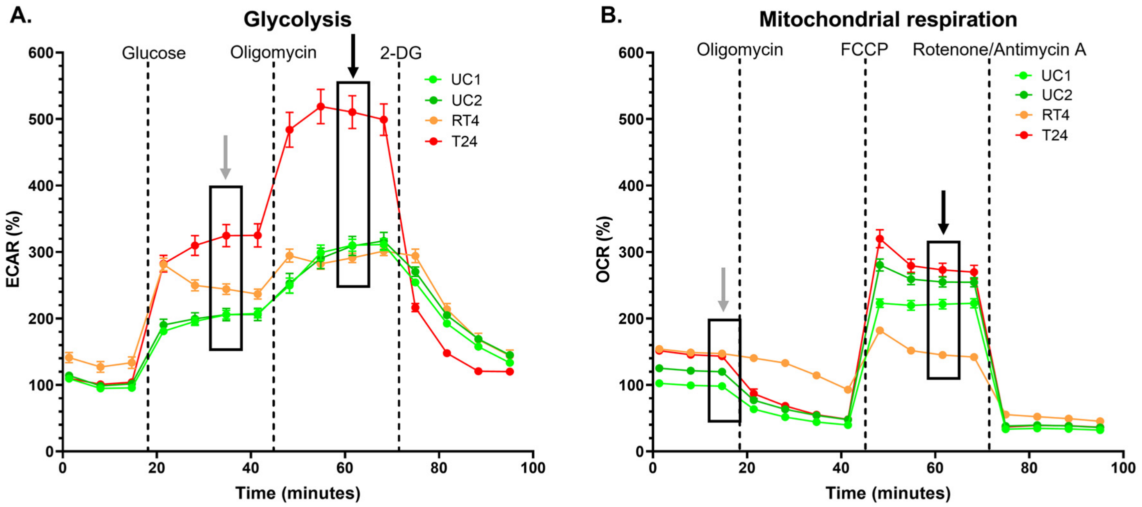

Figure S1. Glycolytic and mitochondrial metabolism of normal urothelial cells (UC1 and UC2) and non-invasive (RT4) and invasive (T24) bladder cancer cells. (**A**) The glycolytic metabolism was established by the sequential injections of glucose, oligomycin and 2-DG. Analyses in Figure 1A-B were performed using measure #6 (gray arrow) for basal glycolysis and measure #10 (black arrow) for maximal glycolytic capacity. (**B**) The mitochondrial respiration was established by sequential injections of oligomycin, FCCP and the combination of rotenone and antimycin A. Analyses in Figure 1C–D were performed using measure #3 (gray arrow) for basal mitochondrial respiration and measure #10 (black arrow) for maximal mitochondrial respiration. Data are displayed as percentages of UC1 acting as control (n = 10, N = 4). The baseline (100%) was established before the first injection, namely before glucose injection for the glycolytic capacity and before oligomycin injection for the mitochondrial respiration.

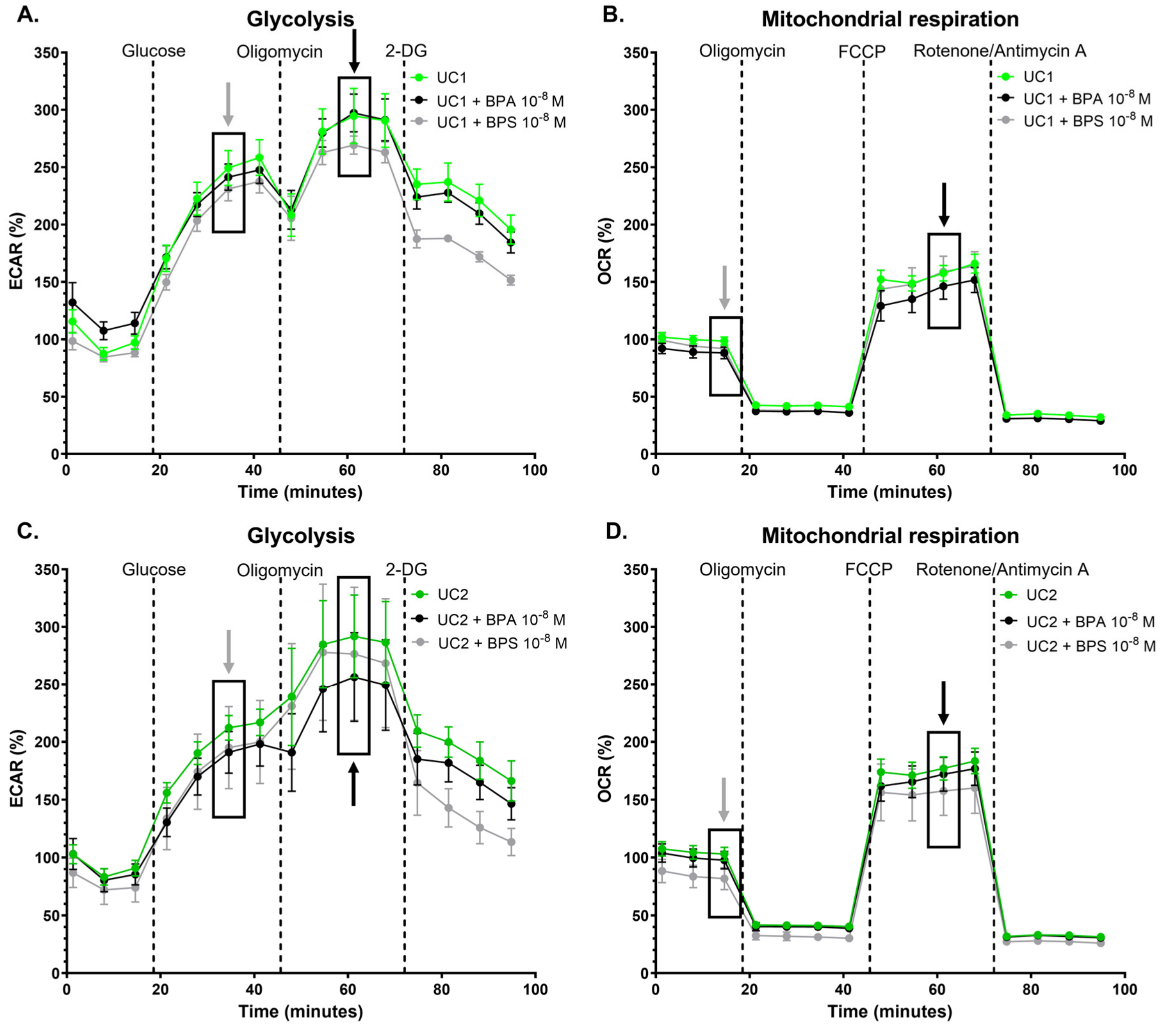

Figure S2. Impact of BPA and BPS on the glycolytic and mitochondrial metabolism of two populations of normal urothelial cells (UC1 and UC2). (**A**,**C**) The glycolytic metabolism was established by the sequential injections of glucose, oligomycin and 2-DG. Analyses in Figure 2 represent the results' combination of UC1 and UC2. Analyses in Figure 2A-B were performed using measure #6 (gray arrow) for basal glycolysis and measure #10 (black arrow) for maximal glycolytic capacity. (**B**,**D**) The mitochondrial respiration was established by sequential injections of oligomycin, FCCP and the combination of rotenone and antimycin A. Analyses in Figure 2**C**,**D** were performed using measure #3 (gray arrow) for basal mitochondrial respiration and measure #10 (black arrow) for maximal mitochondrial respiration. Data are displayed as percentages of controls (i.e., untreated condition) (n = 3, N = 4). The baseline (100%) was established before the first injection, namely before glucose injection for the glycolytic capacity and before oligomycin injection for the mitochondrial respiration.

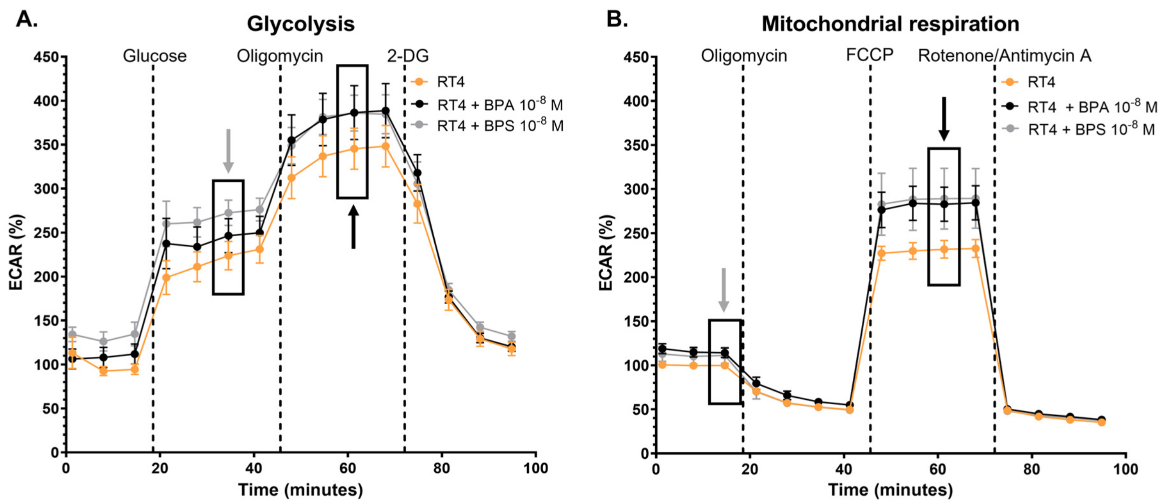

Figure S3. Impact of BPA and BPS on the glycolytic and mitochondrial metabolism of RT4 non-invasive bladder cancer cells. (**A**) The glycolytic metabolism was established by the sequential injections of glucose, oligomycin and 2-DG. Analyses in Figure 3A-B were performed using measure #6 (gray arrow) for basal glycolysis and measure #10 (black arrow) for maximal glycolytic capacity. (**B**) The mitochondrial respiration was established by sequential injections of oligomycin, FCCP and the combination of rotenone and antimycin A. Analyses in Figure 3**C**,**D** were performed using measure #3 (gray arrow) for basal mitochondrial respiration and measure #10 (black arrow) for maximal mitochondrial respiration. Data are displayed as percentages of controls (i.e., untreated condition) (n = 3, N = 3). The baseline (100%) was established before the first injection, namely before glucose injection for the glycolytic capacity and before oligomycin injection for the mitochondrial respiration.

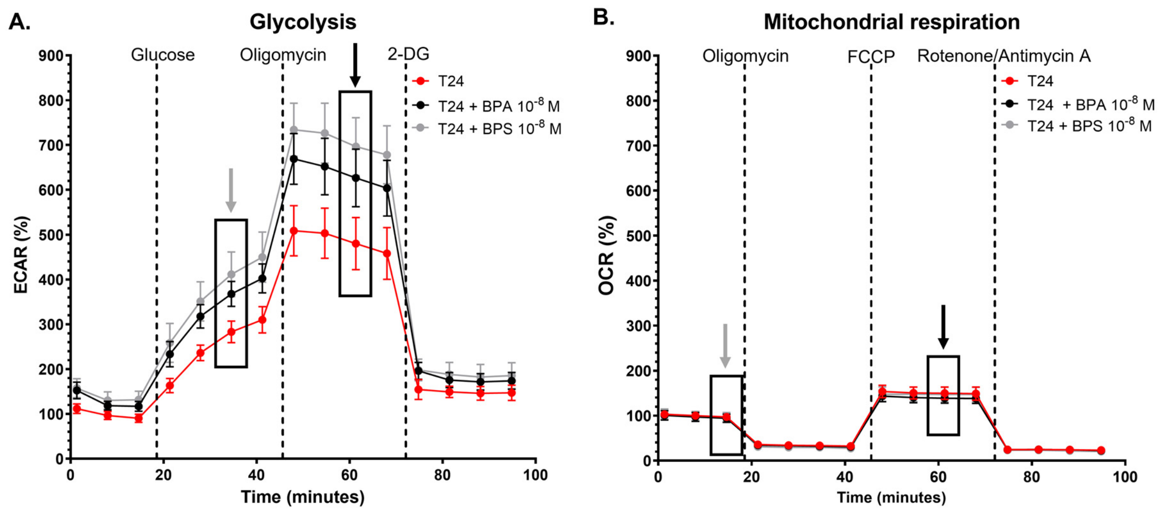

Figure S4. Impact of BPA and BPS on the glycolytic and mitochondrial metabolism of T24 invasive bladder cancer cells. (**A**) The glycolytic metabolism was established by the sequential injections of glucose, oligomycin and 2-DG. Analyses in Figure 4A-B were performed using measure #6 (gray arrow) for basal glycolysis and measure #10 (black arrow) for maximal glycolytic capacity. (**B**) The mitochondrial respiration was established by sequential injections of oligomycin, FCCP and the combination of rotenone and antimycin A. Analyses in Figure 4**C**,**D** were performed using measure #3 (gray arrow) for basal mitochondrial respiration and measure #10 (black arrow) for maximal mitochondrial respiration. Data are displayed as percentages of controls (i.e., untreated condition) (n = 3, N = 3). The baseline (100%) was established before

the first injection, namely before glucose injection for the glycolytic capacity and before oligomycin injection for the mitochondrial respiration.

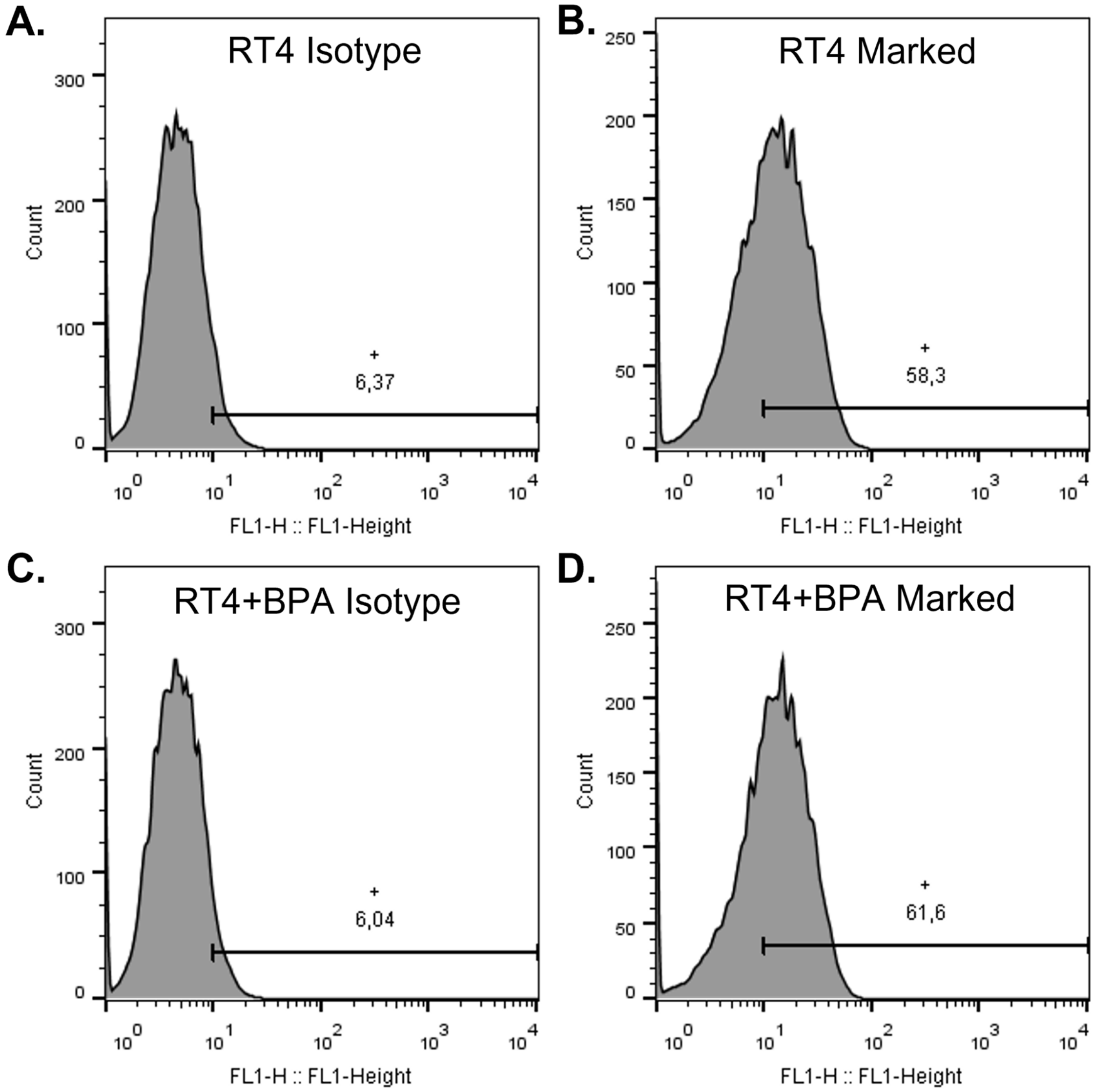

Figure S5. Example of gating analysis for the impact of BPA on the expression of α-SMA of RT4 cells. The gating of α-SMA positive cells was established by subtracting the positive cells marked with the isotype control (**A**,**C**) to the positive cells marked with the anti-α-SMA antibody (**B**,**D**). This example illustrates the gating established for one replicate of RT4 cells (**A**,**B**) and one replicate of RT4 chronically exposed to physiological concentrations of BPA (**C**,**D**).
